# Host cytoskeleton in respiratory syncytial virus assembly and budding

**DOI:** 10.1186/s12985-016-0618-z

**Published:** 2016-09-26

**Authors:** Shadi Shahriari, James Gordon, Reena Ghildyal

**Affiliations:** Respiratory Virology Group, Health Research Institute, Faculty of Education, Science, Technology and Mathematics, University of Canberra, Canberra, 2617 Australia

**Keywords:** Cytoskeleton, Actin, Microfilaments, Molecular motors, Virus assembly, Virus budding, Matrix protein

## Abstract

Respiratory syncytial virus (RSV) is one of the major pathogens responsible for lower respiratory tract infections (LRTI) in young children, the elderly, and the immunosuppressed. Currently, there are no antiviral drugs or vaccines available that effectively target RSV infections, proving a significant challenge in regards to prevention and treatment. An in-depth understanding of the host-virus interactions that underlie assembly and budding would inform new targets for antiviral development.

Current research suggests that the polymerised form of actin, the filamentous or F-actin, plays a role in RSV assembly and budding. Treatment with cytochalasin D, which disrupts F-actin, has been shown to inhibit virus release. In addition, the actin cytoskeleton has been shown to interact with the RSV matrix (M) protein, which plays a central role in RSV assembly. For this reason, the interaction between these two components is hypothesised to facilitate the movement of viral components in the cytoplasm and to the budding site. Despite increases in our knowledge of RSV assembly and budding, M-actin interactions are not well understood. In this review, we discuss the current literature on the role of actin cytoskeleton during assembly and budding of RSV with the aim to integrate disparate studies to build a hypothetical model of the various molecular interactions between actin and RSV M protein that facilitate RSV assembly and budding.

## Background

Respiratory syncytial virus (RSV) is the major cause of lower respiratory tract disease in infants and young children [1] and a major viral agent responsible for respiratory tract disease in immunosuppressed individuals and the elderly [1]. In 2009, the World Health Organisation estimated that globally there are 64 million annual RSV cases leading to 160,000 deaths (http://apps.who.int/vaccine_research/diseases/ari/en/index2.html). Antiviral drugs and vaccines that effectively target RSV infections are currently unavailable, representing a significant challenge in regards to RSV disease prevention and treatment [2]. Virus infection is initiated via inoculation of the nose or eyes which can occur through direct contact or inhalation of airborne infectious particles. This results in viral replication in the nasopharynx over 4–5 days, the virus occasionally spreads to the lower respiratory tract over the next few days [3].

**Fig. 1:**
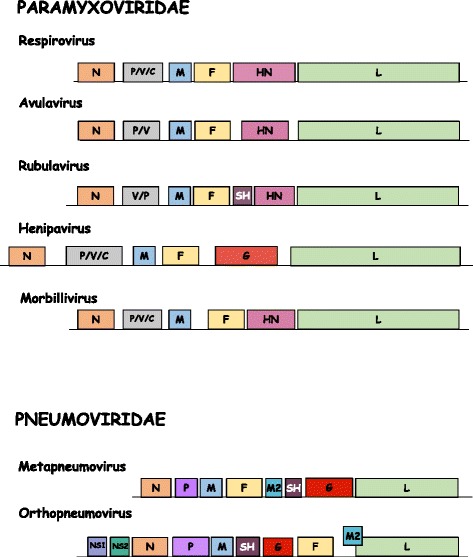
Genome organisation of Paramyxovirus and Pneumovirus genera. The nucleoprotein (N), phosphoprotein (P), matrix (M) protein, fusion (F) protein, and RNA-dependent RNA polymerase (L), are conserved in this order among the viruses belonging to both families. The attachment protein (H, HN, or G), differs amongst the viruses depending on haemagglutinin (H) presence, haemagglutinin-neuraminidase (HN) activity, or neither H present or HN activity (G). Only three genera contain the small hydrophobic SH protein, while both genera belonging to the *Pneumovirinae* subfamily (Pneumovirus and Metapneumovirus) also possess M2, NS1, and NS2 genes. However, gene positions vary between the Pneumovirus and Metapneumovirus genera

RSV is a member of the *Pneumoviridae* family, which consists of two genera, *Metapneumovirius* and *Orthopneumovirus*, with RSV belonging to the latter [4]. The virion is pleomorphic, being either spherical or filamentous [5]. Similar to the closely related Paramyxoviruses, the negative sense RNA genome of RSV is tightly encapsidated within the nucleocapsid, which is composed of nucleocapsid protein N, the RNA polymerase L and its cofactor phosphoprotein P, as well as the M2-1 protein; external to the nucleocapsid is a layer of matrix (M) protein which acts as a bridge between the nucleocapsid and the lipid bilayer envelope. Embedded in the envelope are the fusion (F), large (G) and small hydrophobic (SH) glycoproteins. M2-2 and two non-structural proteins NS1 and NS2 are not found in the virion in any significant amount but have important roles in the RSV replication cycle [5–12]. Figure 1 shows the genome organisation of prototypic members of the Paramyxovirus and Pneumovirus families.

The mechanisms that drive assembly and budding differ among the viruses and thus, no particular mechanism can be assumed for all members. However, in all cases, the M protein drives coordinated interplay between viral structural components to facilitate assembly of infectious progeny. For this reason, M protein is considered as the key organizer of virus assembly [13]. Not only does the M protein interact with viral glycoproteins and the nucleocapsid, it also interacts with the cytoskeleton in infected cells [14], and bundles of actin microfilaments in epithelial cells and fibroblasts are altered during infection. Additionally, similar to members of *Paramyxoviridae* family, RSV has been shown to require actin or tubulin or both for effective transcriptase activity in vitro [15, 16] underscoring the importance of host cell cytoskeleton in Orthopneumovirus lifecycle.

Despite at least two decades of sporadic research investigating cytoskeleton involvement in RSV infection, the role of cytoskeleton in RSV assembly and/or budding is still unclear. In this review we bring together our current knowledge and understanding of the role of the cytoskeleton in RSV infection, drawing on literature on such interactions in other related viruses; the aim being to integrate diverse studies in order to form a rational informed model of RSV-cytoskeleton interactions.

## Virus life cycle

The infectious cycle of RSV follows a similar route to the paramyxovirus lifecycle, however, with several differences, which are detailed in following sections. The paramyxovirus life cycle begins upon attachment of the virus to the host cell and fusion of the viral membrane to the host cell membrane facilitated by the two major glycoproteins, the attachment protein (H, HN, or G depending on the virus; see Fig. 1) and the F protein (Fig. 1). Membrane fusion follows and precedes the release of the viral genome into the cytoplasm [17]. Upon release, the viral genome undergoes primary transcription, followed by translation and replication to form new genomes. The newly synthesised viral genomes are associated with the N protein and wrapped around a nucleocapsid core containing L and P proteins [18]. This process forms the ribonucleoprotein complex (RNPs), which are then transported to the plasma membrane where interaction with the membrane glycoprotein complex takes place, facilitated by the M protein. At the plasma membrane, the RNPs, the M protein, and the glycoproteins form the paramyxovirus particles, which are then released through membrane scission [13].

### RSV entry

**Fig. 2:**
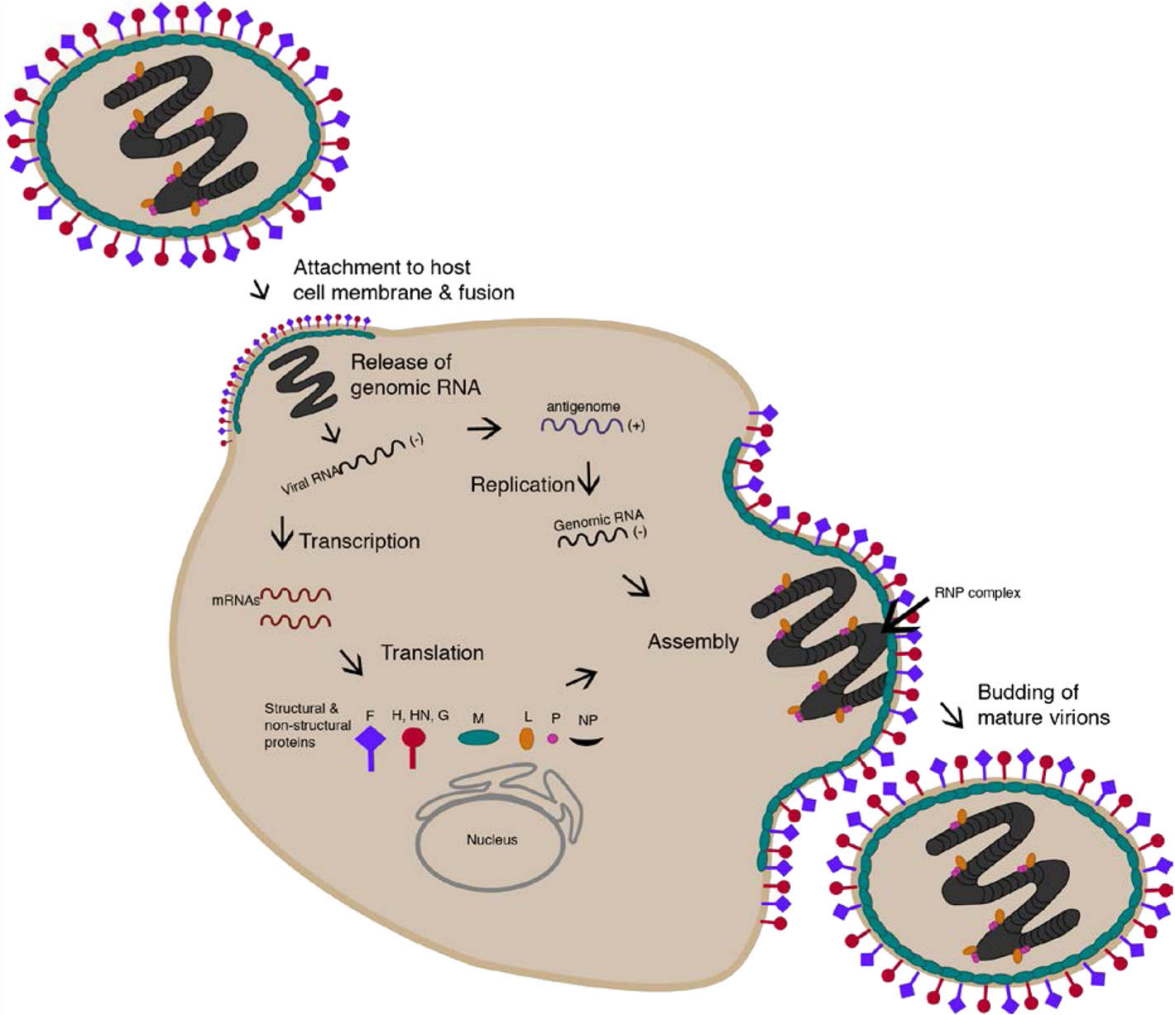
Paramyxovirus Lifecycle. Attachment to the host cell membrane initiates the viral lifecycle and entry occurs by the release of the viral genome into the cytoplasm. The negative-sense RNA genome undergoes primary transcription to produce mRNAs. The viral genome is replicated in a two-step process catalyzed by the viral polymerase (composed of phosphoprotein (P) and large (L) protein components) where antigenome intermediates are produced from genomic templates before the production of negative-sense genomes. The helical nucleocapsid is formed by association of the newly synthesised nucleoprotein (NP) with the nascent genomic RNA. This newly formed structure then interacts with the RNA-dependent RNA polymerase complex (RNPs). RNPs are transported to the plasma membrane by M protein where they interact with surface glycoproteins for assembly, before membrane scission and release of virus particles. The figure was generated using Adobe Illustrator

Of the three RSV envelope glycoproteins, only F is essential for attachment and entry although G deleted strains are highly attenuated in vivo [19]. Glycosaminoglycans (GAGs) are cell surface receptors implicated in RSV attachment in cell culture, as well for many other viruses [20, 21]. Interestingly, nucleolin has been shown to function as an entry receptor for RSV via F glycoprotein; non-permissive cells become susceptible to RSV infection when transfected to express human nucleolin [22]. As nucleolin is ubiquitously expressed, there is probably another receptor that determines tropism. Of direct relevance to this review is the fact that cell surface nucleolin mediates internalisation of ligands via its interaction with the actin cytoskeleton [23]. Entry of RSV genome has been described as F mediated fusion of viral envelope and host cell membrane (Fig. 2), though some evidence implicates clathrin mediated endocytosis in the entry process, independent of endosomal acidification [24, 25].

### RSV transcription and translation

The minimum protein requirement for RSV transcription is N, P and L. In infected cells, these proteins are found in cytoplasmic inclusions, which also contain viral RNA of both polarities and are presumed to be sites of transcription [26]; recent studies suggest that inclusions are not structurally or functionally homogenous [27] being implicated in innate response to RSV infection. Transcription results in 10 capped, methylated and polyadenylated mRNAs which are translated by host cell machinery. Each gene is flanked by a gene-start (GS) and gene-end (GE) sequence [28, 29]. A promoter at the 3’ end of the genome acts as the only attachment site for the polymerase complex [5]. Genes are then transcribed sequentially from 3’ to 5’ end. However, due to polymerase drop off at intergenic regions, there is a gradient of expression, with genes at the 3’ end expressed at higher levels than genes at the 5’ end. Hence, proteins that the virus needs early and in greater numbers such as the interferon antagonists, non-structural proteins NS1 and NS2, are produced much more than proteins needed in small supply such as the polymerase L protein. The end of the M2 gene and start of the L gene overlap by 68 nucleotides. The polymerase complex overcomes this difficulty by backtracking to the start of the L gene after completing transcription of M2 [30]. This means that to complete transcription of L, the polymerase complex must “read through” the GE sequence of M2, something that it does not do in 90 % of cases [5]. The benefit of this added complexity, if any, is uncertain. The M2 gene also has the distinction of coding an additional protein via a second, slightly overlapping open reading frame. The M2-2 protein thus produced, has a non-structural function and is dispensable for RSV replication [31].

### RSV replication

Replication of new negative sense genomes requires the production of a complete positive sense anti-genome as an intermediate. This is accomplished with the polymerase complex used for mRNA transcription but operating in “read through” mode where GS and GE signals are not recognised. The switch from transcription mode to replication mode is of great interest to researchers but little is known of this mechanism aside from M2-2 being implicated. In cells infected with RSV without M2-2, the level of viral mRNA was increased while genomic RNA was decreased [31].

### RSV assembly

Glycoproteins are translated and traffic through the secretory pathway to the apical surface, while internal virion proteins remain in the cytoplasm. Generation of nascent RSV genomic RNA probably occurs in cytoplasmic inclusions. N and P form RNPs that associate with the M protein, then traffic to the apical cell surface where they meet the glycoproteins. At the cell surface, viral proteins assemble into viral filaments that contain both viral structural proteins and viral genomic RNA. These filaments are thought to assemble into infectious virion particles prior to separation from the cell surface, since they can contribute to cell-cell spread of the virus and morphologically resemble the filamentous form of virions seen in electron micrograph studies of virus produced in polarized cells [5].

Although significant advances have been made in our understanding of RSV assembly and elucidation of the interactions between M and other viral proteins, our current knowledge on how the RNPs and the envelope glycoprotein complexes move from their cytoplasmic location to the plasma membrane is still limited. Literature from work with other viruses suggests a possible role for the cytoskeleton in this movement. Specifically, proteins associated with the actin network are implicated in RSV budding [32–34].

### RSV budding

As mentioned previously, RSV infection is restricted to the superficial cells of the respiratory epithelium. In keeping with this, budding in polarised epithelial cells occurs at the apical membrane [35, 36] and preferentially from lipid rafts [37, 38]. Viral budding occurs in a Vps-4, ESCRT pathway independent manner resulting in pleomorphic particles that can be spherical or filamentous [39]. Recent literature suggests that RSV proteins are the main drivers of the formation of filamentous virus, with cytoskeletal proteins playing a supporting role [40].

It is likely that most, if not all, viruses utilise the cytoskeleton in some way. The most compelling argument to support this is that while salts and gases are small enough to diffuse through the cytoplasm, larger entities such as mature virions and large subviral particles must utilise the cytoskeleton transport system for active transport [41]. Support for this idea comes from work that examines the viscosity and diffusion properties of the cytoplasm. It was found that the diffusion of hydrophilic, electroneutral beads of 80 nm in radius was between 500 and 1000 fold lower in the cytoplasm than in aqueous solution [42]. Data from the same study has been used to estimate that vaccinia virus would take 5 h to diffuse a mere 10 μm in the cytosol [43]; however vaccinia virus movement from the juxtanuclear region to the periphery is significantly faster (>2 μm/s).

In the following sections we provide a brief overview of the cytoskeleton, followed by examples of its exploitation by chosen viruses; finally, we present current literature on the role of cytoskeleton in RSV assembly and budding and possible interactions with M protein.

## The cytoskeleton

The cytoskeleton is composed of three different types of protein filaments which in order of smallest to largest are (filamentous) actin, intermediate filaments and microtubules. Apart from being the scaffolding that gives the cell its shape and strength, the cytoskeleton has other important functions. Not only does the cytoskeleton play a role in positioning and holding organelles in place, it also allows for cell locomotion. In addition, events such as cytokinesis and the movement of chromosomes during anaphase rely on the cytoskeleton and its associated molecular motors [44].

### Actin

Actin is the most abundant cytoskeletal protein in eukaryotes and typically accounts for 5 to 10 % of total protein [44]. Actin monomers (G-actin) assemble into filaments (F-actin) with a distinct polarity; they have a fast growing barbed end and a slow growing pointed end [45]. The implication of this polarity is that when used as a track for intracellular transport, motor proteins only carry cargo in a specific direction. Actin filaments can be arranged into a variety of linear bundles and two-dimensional arrays, or three-dimensional gels [46]. Numerous actin binding proteins regulate the polymerisation and organisation of F-actin and it is these proteins that are sometimes the target of viral manipulation [32]. Although actin is present throughout the cytoplasm it is most abundant at the periphery of the cell which is where polar actin networks such as filopodia, lamellipodia and pseudopodia reside [47]. The peripheral actin network is sometimes referred to as the actin cortex. The actin cortex is generally arranged with plus ends facing outward and is the key player in cell motility which is based on filament assembly and the action of myosins [48]. The actin cortex has been proposed to be an obstacle to viral entry [49] and this might be why some viruses use the endocytic pathway which easily crosses the actin cortex [46]. Regulated binding to actin filaments can control the localisation of viral proteins such as the nucleoprotein of influenza virus [50].

### Microtubules

Microtubules are the largest of the cytoskeleton filaments and like actin filaments they display a distinct polarity as well as a fast growing end and a slow growing end. Microtubules are composed of dimers of α-tubulin and β-tubulin arranged head to tail lengthwise and 13 side by side around a hollow core [44]. Most of the microtubules in a cell radiate outward from a microtubule organising centre (MTOC), also called a centrosome which is usually located near the nucleus. Microtubules extend from the MTOC with plus ends facing outward. Different cell types such as polarised epithelial cells and neurons can have specialised microtubule networks. Microtubules in axons are longitudinally arranged with plus ends pointing toward the axon terminal and minus end pointing toward the soma [51] while in polarised epithelial cells microtubules are arranged with plus ends pointing toward basal membrane and minus ends pointing toward the apical membrane [46]. The main role of microtubules in viral infection is to provide the tracks that the motor proteins kinesin and dynein use for long range transport of viral proteins, nucleocapsids and enveloped virions. This role has been described previously in many reviews [41, 52, 53], and will be described briefly in this review.

### Intermediate filaments

Intermediate filaments have several important differences to F-actin and microtubules. They exhibit no treadmilling behaviour and have no polarity [44]. The general role of intermediate filaments is more of a structural support role and to provide scaffolding for the localisation of cellular processes, especially the nucleus, around which intermediate filaments form a ring. They are composed of over 65 different proteins enabling intermediate filaments to be turned to a variety of different purposes [44]. Intermediate filaments are known to be disassembled or rearranged by viral proteases [54, 55]. This may serve the virus by removing a barrier to free diffusion or it may weaken the cell enough to enhance cell lysis and release of viral progeny.

### Molecular motors

Motor proteins use ATP hydrolysis to power their movement along cytoskeleton filaments. If the motor protein is fixed, it moves the filament and if the motor protein is not fixed, it moves cargo along the filament. Myosins transport cargo along actin filaments, whereas kinesins and dynein move along microtubules. There are 18 classes of myosin, grouped according to homologous sequences in the tail domain which determines cargo specificity. The most well-known and best studied is myosin II, the myosin responsible for muscle contraction. Most classes of myosin are plus end directed motors with the exception of myosin VI and possibly IXb, which are minus end directed [46]. Kinesins and dyneins transport membrane vesicles, macromolecules, organelles and viral particles/proteins toward the plus end and minus end, respectively, of microtubules.

## The cytoskeleton in virus assembly

The host cell cytoskeleton plays an important role in assembly and budding of several viruses across different families.

### Vaccinia virus

Vaccinia virus is a large double stranded DNA virus that exploits actin and tubulin cytoskeletons to ensure that its components are at the right place at the right time for optimal infectious virus production. After entry, viral cores use microtubules to move centrally where viral gene expression and formation of intracellular mature virus (IMV) occur [56]. The IMVs now use microtubules to move from viral factories to areas near the MTOC where they acquire an envelope from the *trans*-Golgi network or early endosomes and become intracellular enveloped virions (IEV) [57]. IEV is the intermediate between IMV and cell-associated enveloped virions (CEV). After acquiring the envelope, IEVs again use microtubules but this time in the opposite direction as they travel toward the periphery where they fuse with the plasma membrane and are exposed on the outside of the plasma membrane as CEV [58]. The next stage of the vaccinia lifecycle illustrates an impressive subversion of the actin cytoskeleton by the virus. CEV are able to induce the polymerisation of actin, creating protrusions known as actin tails that facilitate cell to cell spread by pushing the exposed CEV into adjacent cells [59].

### Poliovirus

The positive sense RNA virus Poliovirus exploits the actin cytoskeleton during infection; after binding, poliovirus is internalized through an actin dependent pathway followed by rapid release of the RNA [60]. Interestingly, poliovirus was shown to move rapidly in the cytoplasm with unusually high speeds, independent of microtubules, but both ATP- and actin-dependent, suggesting an active transport mechanism. However, the viral trajectory and speed could not be accounted for by transport along microfilaments and a model wherein poliovirus induces changes in the activity of motor proteins was proposed [61].

### Herpesvirus

Neurotropic viruses such as herpes simplex viruses (HSV) need to travel long distances within neurons. HSV takes control over the cytoskeleton early in infection by remodelling the cortical actin cytoskeleton [62]. The purpose of this remodelling may be to prepare the cell for efficient exit of virus later in infection [63]. Viral capsids interact directly with dynein [64], in order to travel the long distance to reach the nucleus. After entering the nucleus, HSV genomes are transcribed, replicated and packaged into new capsids. HSV induces the formation of actin in the nucleus even in cells where there is normally no nuclear actin [65]. Newly formed capsids recruit myosin for active transport along these actin filaments to the periphery of the nucleus [66]. Several HSV proteins have been shown to induce conformational changes in the nuclear lamina that may facilitate nuclear egress [67, 68]. Virions then travel back down the axon to the site of exit via microtubule transport through the recruitment of kinesin [52].

## Cytoskeleton in paramyxovirus, pneumovirus life cycle

### Entry

The host actin cytoskeleton plays an important role in the life cycle of paramyxoviruses [15, 69, 70]. The actin network creates a physical barrier against the invading virus, which must be overcome to enable productive infection [71]. This is made possible by the remodelling of the actin skeleton that is stimulated during paramyxovirus (and RSV) infection. Bundles of microfilaments are established through the formation of stress fibres [71] regulated by the Rho-family of GTPases, which play a role in controlling signal transduction pathways that generate changes in the actin cytoskeleton [71, 72] and are involved in influencing the assembly or disassembly of F-actin [73].

### Assembly

Several paramyxoviruses, including Sendai (SeV) and measles virus (MeV), utilise vesicles that contain key regulators of microtubule transport such as Rab11A. The apical recycling endosome (ARE) pathway, that Rab11 endosomes are associated with, is important for the transport of proteins in polarized cells [13, 39, 74]. Although this notion suggests that Rab11A is required only in polarized cells, it has been demonstrated that the requirement is for both polarized and non-polarised cells with regards to SeV assembly [39]. In MeV infection, Rab11A is required only for virus production in polarized cells and not for RNP transport [75]. The presence of a dominant-negative form of the Rab11-interacting protein myosin Vb (MVb), results in reduced RSV assembly at the apical membrane. Of interest is Rab11-FIP2 (FIP2), another Rab11 interacting protein that may be involved in formation of filamentous virions [39]. Parainfluenza virus 3 (hPIV3) requires microfilaments for replication; however, its assembly is dependent on microtubules. This is in contrast to SeV and MeV where assembly depends on microfilaments as well as microtubules [75].

### Budding

Cytoskeletal components are also involved in the budding of parvirus particles [13, 76, 77]. Actin involvement in budding was first discovered through studies undertaken on SeV and MeV particles, where large quantities of actin were detected in purified preparations of the two viruses. Studies on SeV have suggested that expression of M or F allows for the production of virus like particles (VLPs) that contain actin; both M and F proteins are required for budding. Significantly, sequences within M and F proteins resemble actin-binding domains [78, 79]. Mutations in the actin binding domains of SeV F result in significant reduction in VLP production suggesting that SeV F protein binding to actin is required for budding to take place [13].

Cytochalasin D, a specific inhibitor of actin polymerisation, inhibits release and reduces infectivity as well as entry of paramyxoviruses [71, 75]. As the microfilament network plays a role in the trafficking of RNP complexes, it is not surprising that virus release is affected by such drugs [13, 71]. However, treatment of hPIV3 infected A549 alveolar carcinoma lung cells with cytochalasin D had no effect on virus release. In contrast, microtubule depolymerisation drugs seemed to have more of a inhibitory effect on hPIV3 virus release [75]. Therefore, it is evident that each virus utilizes the cytoskeleton differently, however, the cytoskeleton does play an important role in replication, assembly, transportation, and budding of paramyxoviruses (Table 1).

**Table 1:** Requirement of cytoskeletal components in paramyxovirus replication and assembly

Virus	Cytoskeletal component	Virus lifecycle stage	Reference
Measles virus	Tubulin	Replication	[76, 95]
	Microfilaments	Budding	
Sendai virus	Actin	Transcription	[71, 76]
	Tubulin	Transcription	
	Microfilaments	Assembly	
Respiratory syncytial virus	Actin	Transcription	[33, 82, 84, 96, 97]
	Tubulin	Transcription	
	Microfilaments	Entry, Assembly	
Human Parainfluenza virus type 3, 5	Actin	Transcription	[98, 99]
	Microfilaments	Assembly	

### Paramyxovirus M protein and cytoskeleton

The interaction of Newcastle disease virus (NDV) and SeV M proteins with actin filaments is important during virion maturation [70]. Expression of SeV M protein is essential for the actin restructuring that is observed during infection and virion release. MeV M-RNP complexes use the F-actin network to move to the plasma membrane; however, actin dynamics are required for viral budding [69].

## Cytoskeleton in RSV infection

RSV is known to interact with the cytoskeleton in several ways and at various stages throughout its life cycle. In addition to transcriptional regulation, the cytoskeleton is also likely to be involved in the directional movement of RSV components. Live video microscopy showing filamentous virus budding from the same region of cell membrane at a rate of several virions per minute suggests directed transport [80]. The picture that has emerged so far seems to be that of the three cytoskeleton components, actin is the most important in RSV pathogenesis. However, the microtubule inhibitor nocodazole has been shown to reduce RSV titre by almost as much as cytochalasin D (9 fold reduction compared to 10 fold reduction) [81]. In the following sections, we present an overview of the known interactions, focussing on M and F proteins.

### Transcription

Actin is present as an internal component in purified RSV virions [34] and antibodies against actin inhibit RSV transcription [33]. In a study using microarrays to analyse host gene expression in RSV infected cells, it was found that five genes associated with the cytoskeleton were upregulated shortly after infection [82]. There is evidence that RSV is indirectly involved with the cytoskeleton through interaction with actin associated proteins. Profilin, an actin-modulatory protein, has a significant role in RSV transcription [15, 32]. Although the RSV polymerase complex is able to mediate transcription in the absence of any cellular factors, actin is required for optimal activity. Current understanding of paramyxovirus transcription suggests that monomeric actin is required for transcriptase activity; the presence of actin in the virions likely reflects the involvement of actin in RSV transcription [75].

### Assembly

The cytoskeleton has been suggested to play a role in transport of RNPs to RSV budding sites; RNPs display a myosin motor driven directional movement on the actin cytoskeleton [77]. Using live cell tracking of RNPs in cells infected with a recombinant RSV that expressed GFP, Santangelo and Bao [77] showed that RNPs move from the vicinity of filamentous virus at the apical surface and formed circular, inclusion-like structures in the cytoplasm on treatment with cytochalasin D. Importantly, myosin colocalised strongly with the virus filaments but not with cytoplasmic inclusion-like structures; suggesting myosin’s role in movement of RNPs to the budding site. Given the key role of M and actin in RSV assembly, it is important to understand the interactions between M and actin which underlie M’s role as facilitator and bringing together the RNP and envelope glycoproteins for virus assembly at the plasma membrane.

### Budding

That microfilaments are required for optimal RSV budding and release is shown by the fact that virus release is inhibited by the microfilament destabilizing drug cytochalasin D, as mentioned previously [34, 75, 83].

In view of the connection between lipid rafts and the actin network [84], and that RSV buds from lipid raft domains of the plasma membrane [37, 85], myosin-driven transport likely also plays a role in RSV budding and release [77]. Increased F-actin is also observed at the lipid raft domain budding sites of human metapneumovirus [86], suggesting assembly/budding mechanism similar to RSV. Actin-modulatory proteins profilin and RhoGTPase are associated with RSV budding. As an actin-modulatory protein, profilin plays a role in promoting the formation and stabilization of stress fibres. In RSV infected cells, it has a role in virion morphogenesis and cell fusion in addition to enhancing the transcriptase activity [71]. RhoA GTPase has a variety of functions in the cell, including organisation of the actin cytoskeleton; blocking its action has a deleterious effect on RSV budding [87, 88] and RSV infection results in activation of RhoA, leading to rearrangement of the actin cytoskeleton. Interestingly, inhibition of RhoA leads to virus filaments adopting a more spherical particle morphology rather than a blunted one [89]. Phosphoinositide-3-kinase (PI3K) signalling, which has an important role in activation of F-actin associated signalling pathways that lead to structural changes in the F-actin network during infection, has a role in the organisation of RSV proteins at assembly sites [83]. In addition, PI3K activity may be involved in the formation of pneumovirus filaments that enable cell-to-cell viral spread [71, 89].

Although cytoskeletal components are clearly associated with RSV assembly and budding, studies undertaken on the effects of actin or tubulin disruption have suggested that RSV may assemble into filaments using a mechanism that does not depend on the host cytoskeleton [89].

### RSV F protein

The F protein is essential for assembly and budding of RSV. The F-protein cytoplasmic tail (FCT) has been shown to be required for efficient replication as studies on viruses lacking this domain have effectively demonstrated a 100- to 1000-fold decrease in viral titres. RSV infection induces the formation of filaments, which have been shown to contain the viral proteins F, M, N, and P [89]. The formation of RSV filaments appears to depend on the FCT, especially a specific phenyalanine (Phe) residue at position 22 [13, 89, 90]. Interestingly, a mutation in Phe^22^ leads to the inability of RSV F to recruit viral proteins into VLPs and, thus, form filaments [89]. Therefore, the F protein plays a role in coordinating the assembly of the virus filaments at the cell surface [13, 89, 90].

The F protein is key to the formation of syncytia that are characteristic of RSV infection. F interacts directly with RhoA in vitro and inhibition of this interaction in infected cells results in abrogation of syncytia formation [39]; clearly, RhoA-F interaction drives cell to cell spread of RSV.

### RSV M and F protein

The minimum RSV assembly complex contains F, M and the RNP core [91] and is formed in cytoplasmic inclusions [90]. Although cytoplasmic inclusions are characteristic of RSV infected cells, their role in infection is not completely understood [27, 83, 89]. It has been suggested that inclusions are sites of replication and/or transcription, sites for dead-end product accumulation, or sites for morphogenic intermediates [27]. RSV M protein associates with cytoplasmic inclusions late in infection through its interaction with the M2-1 protein and has been suggested to play a role in the switch between transcription and assembly [92, 93]. One study has effectively shown that both M and F protein association with cytoplasmic inclusions is critical in the assembly process, a step preceding filamentous virion formation, supporting the idea that viral filaments are formed or initiated from inclusions acting as scaffolds [90]. These associations with inclusions have been shown to be related to FCT-dependent changes, where increased amounts of F protein accumulated at the inclusions in the absence of FCT. In addition, the proportion of M associated with inclusions has been shown to be low when there is an abundance of filamentous (as opposed to spherical) virus. In turn, the M is increased in the cytoplasm in studies that involved the deletion of FCT, suggesting that the cytoplasmic M requires association with inclusions in order for incorporation into filaments [90].

F protein targets virus components to the lipid rafts, sites of virus budding [39, 89]. F and G glycoproteins as well as M are sorted into lipid rafts in RSV infected cells [38], with M forming structures suggesting an interaction with the cytoskeleton [89]. Inhibition of the Golgi secreting pathway prevents M being sorted into lipid rafts. M and F are co-localised to lipid rafts in cells co-expressing the two proteins and in RSV infected cells.

### RSV M protein and actin

The M protein has been suggested to interact with actin for assembly and budding of RSV virions [94]. Both the M protein and the actin cytoskeleton have previously been described to individually play a role in the assembly of RSV. As mentioned, the M protein interacts with the RNP complex to inhibit the transcriptase activity probably in preparation for packaging [92]. As M also interacts with the host cytoskeleton, studies have suggested that there is an important role for this cellular component in RNP transport to the plasma membrane [77]. Current knowledge suggests that the M-containing complex is anchored onto the microfilament network that the nucleocapsid takes advantage of to reach RSV budding sites [89, 90]. The formation of filamentous virus is probably mediated through the interaction between RNPs, envelope glycoprotein complex and the plasma membrane by association of M with FCT [6, 37].

## Conclusions: proposed model for the role of actin in RSV assembly and budding

**Fig. 3:**
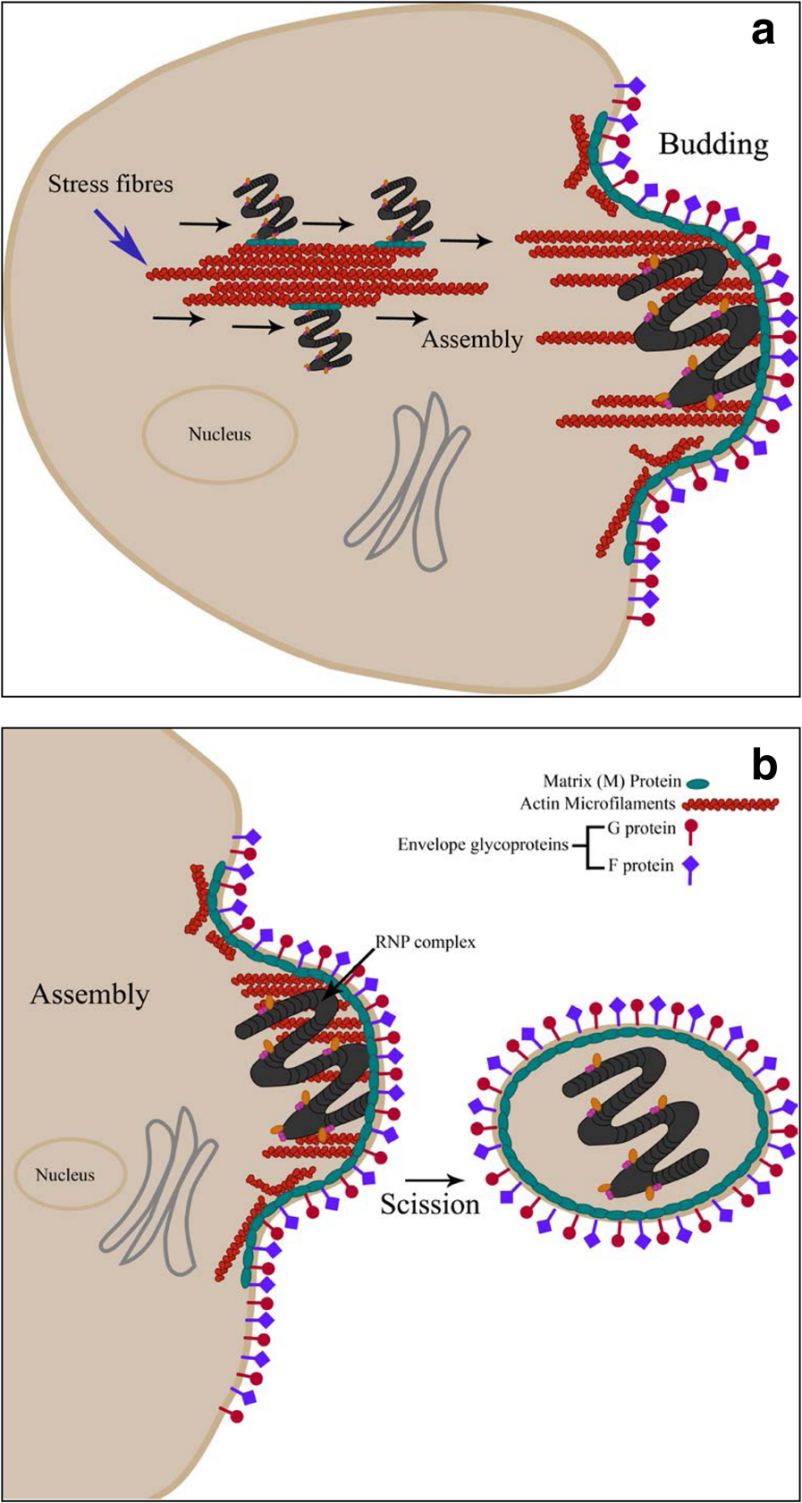
Model for RSV use of microfilaments in viral assembly. **a** The M-containing RNPs of RSV utilise microfilaments (stress fibres) as a means of transport to reach assembly sites; this movement is facilitated by the M protein interaction with microfilaments. At the assembly site, the RNPs associate with bundles of actin filaments and the envelope glycoproteins to complete viral assembly and initiate budding. **b** The growth of the viral filament is supported by microfilaments and actin-modulatory proteins; mature virions are released through as yet unknown mechanism of membrane scission

Based on current literature on RSV as discussed above, it appears that the RNPs of RSV use microfilaments as a means of transport to reach assembly sites as shown for the closely related paramyxoviruses (Fig. 3); this movement may be mediated by M’s interaction with microfilaments, ensuring that only mature RNPs move to the plasma membrane. F protein probably also associates with the complex at this time mediated by M-FCT interaction to target the complex to the lipid rafts. At the cell surface assembly site, M-RNPs (+/− F) may associate with envelope glycoproteins via M’s self-oligomerising activity that allows it to bind to the M-F/G complexes (formed through M’s interactions with cytoplasmic tail of G, and F) forming the budding particle. FCT functions to engage RhoA, leading to extension of the virus filaments, followed by an as yet unknown scission mechanism that releases the RSV particles.

## Abbreviations

ARE: Apical recycling endosome
ATP: Adenosine triphosphate
CEV: Cell-associated enveloped virions
ESCRT: Endosomal sorting complexes required for transport
F-actin: Filamentous actin
FCT: F-protein cytoplasmic tail
G-actin: Globular actin
GAGs: Glycosaminoglycans
GFP: Green fluorescent protein
hPIV3: Parainfluenza virus 3
HSV: Herpes simplex virus
IEV: Intracellular enveloped virions
IMV: Intracellular mature virus
LRTI: Lower respiratory tract infection
MeV: Measles virus
MTOC: Microtubule organizing centre
MVb: Myosin Vb
NDV: Newcastle disease virus
PI3K: Phosphoinosulfide-3-kinase
Rab11A: Ras-related protein
Rab11A-FIP2: Rab11 family-interacting protein 2
RNPs: Ribonucleoprotein complexes
RSV: Respiratory syncytial virus
SeV: Sendai virus
VLPs: Virus-like particles
